# Neuromodulation With Electromagnetic Field Stimulation via Gamma Oscillations Improved Mini-Mental State Examination Scores in Patients With Cognitive Impairment

**DOI:** 10.7759/cureus.101122

**Published:** 2026-01-08

**Authors:** Alice S Wang, Maxwell Marino, Andrew Helson, Mohammad Hossein Abbasi, Jessica Dally, Dan E Miulli

**Affiliations:** 1 Neurological Surgery, Riverside University Health System Medical Center, Moreno Valley, USA; 2 Neurological Surgery, Arrowhead Regional Medical Center, Colton, USA; 3 Neurology, Arrowhead Regional Medical Center, Colton, USA; 4 Neurological Surgery, California University of Science and Medicine, Colton, USA

**Keywords:** cognitive impairment and dementia, electromagnetic field stimulation, mini-mental state examination, neuromodulation therapies, noninvasive brain stimulation

## Abstract

Background: Abnormalities in gamma oscillations have been found in neurological disorders that involve dementia, such as Alzheimer’s disease. Neuromodulation via gamma stimulation has shown promising potential to enhance cognitive function in patients with Alzheimer’s disease.

Objective: In this pilot clinical trial, we describe recording and neuromodulation of brain electromagnetic field (EMF) at gamma oscillations, specifically 70 Hertz (Hz) to 100 Hz, and its effect on brain EMF waves, cognition, and memory as assessed with EMF recordings and Mini-Mental State Examination (MMSE) in patients with cognitive impairment. We hypothesize that EMF stimulation at 70-100 Hz will lead to a statistically significant improvement in MMSE compared with baseline. Additionally, we aim to refine EMF recording and stimulation protocols.

Methods: MMSE was performed before and after EMF recordings. We used a previously developed portable helmet system equipped with Mu-metal (MuMETAL, Magnetic Shield Corporation, Bensenville, IL) and copper shielding, embedded sensors, and EMF generators to record baseline brain EMF of patients with cognitive impairment, identify the sensor of interest and frequency of interest, deliver EMF stimulation at the frequency of interest at 10 Volts over 10 minutes, and record post-stimulation EMF.

Results: Sixteen patients with cognitive impairment were included in this study. EMF recordings from six patients were used to refine analysis protocols, while ten new patients underwent stimulation. The mean pre-stimulation MMSE score was 13.8/30 points, and the mean post-stimulation MMSE score was 17.5/30 points (p=0.170).

Conclusions: In this pilot study, neuromodulation via EMF stimulation led to improvement in EMF waves and showed a trend toward cognitive and memory improvement without statistical significance in patients with cognitive impairment.

## Introduction

Cognitive impairment is on the rise, given the global aging population, with Alzheimer's disease (AD) as the primary cause [1]. Among the many cognitive screening tools, the Mini-Mental State Examination (MMSE) is a popular and commonly used set of standardized questions that are easy to perform. A score of 24-30 is considered normal cognition, 18-23 mild cognitive impairment, and 0-17 severe cognitive impairment [2].

Many studies have investigated cognitive functions tested in the MMSE to corresponding brain areas: orientation to time and place (frontal lobe and temporal lobe), orientation to person (medial prefrontal cortex, posterior cingulate cortex, fusiform gyrus), immediate recall (Wernicke, Broca, arcuate fasciculus), delayed recall (hippocampus, medial temporal lobe), attention in spelling (prefrontal cortex, frontal dorsolateral cortex, inferior parietal cortex, cingulate gyrus), attention in calculation (prefrontal cortex, frontal dorsolateral cortex, left parietal cortex, cingulate gyrus), attention lacking perseveration (frontal lobe), language repetition (Wernicke’s, Broca's, fasciculus arcuatus), language three-step command (temporal, frontal, premotor), language reading and comprehension (left parietal cortex, temporal cortex), language writing (left parietal lobe), copy design (right parietal (construct, gestalt), basal ganglia with projections to the prefrontal cortex), verbal fluency (frontal cortex, prefrontal cortex), right-left orientation (left parietal cortex), praxis in writing (left parietal lobe), and visual processing (occipital cortex) [3].

Newer technology using voxel-based morphometry, for example, has found lower MMSE scores are associated with reduced gray matter volume in the bilateral lateral frontal lobes, left medial frontal lobe, dorsolateral prefrontal cortex, medial temporal lobe, bilateral hippocampus, entorhinal cortex, left parahippocampal gyrus, temporoparietal cortex, left anterior cingulate cortex, bilateral inferior temporal gyri, right ventromedial prefrontal cortex, occipital cortex, and precuneus [4-7]. Furthermore, the MMSE scores are inversely correlated to total gray matter volume atrophy with a preference in the limbic system, including the hippocampus, amygdala, and parahippocampal gyrus, all localized to the temporal region [7]. The extent of gray matter volume atrophy extends beyond these areas into the Papez circuit, which regulates memory function. The medial prefrontal cortex and lateral prefrontal cortex, which are involved in cognitive control, and posterior medial (precuneus) and lateral (temporal gyrus) regions, which are involved in visuospatial imagery and consciousness, also show gray matter volume reductions [8,9]. Additional areas are also found to be associated with cognitive impairments: bilateral caudate nucleus, bilateral insula, left superior parietal lobule, and angular gyrus [10].

By studying patients with normal cognition, mild cognitive impairment, and Alzheimer's disease, the pattern of region-specific gray matter atrophy has been mapped out. Early degeneration is first observed in the medial temporal structures, such as the hippocampus and parahippocampal gyrus, in patients with normal cognition and mild cognitive impairment, followed by more extensive cortical areas, such as the inferior temporal gyrus, in patients with mild cognitive impairment and Alzheimer's disease [4,6-7].

On the cellular level, communication among neurons generates electromagnetic fields (EMFs) that can be measured as brainwaves, which are categorized into different types based on their frequencies. One type is gamma oscillations, ranging from 25 Hertz (Hz) to 100 Hz. They are important in perception, movement, memory, and emotion [11]. They are seen in the hippocampus, which may be involved in attentional selection and memory operations, and other brain regions, including the entorhinal cortex, prefrontal cortex, amygdala, basal ganglia, and thalamus [12,13]. The gamma waves are further divided into slow (25-50 Hz) and fast (55-100 Hz) [14]. The slow gamma oscillations receive input from CA3 to CA1, and these are involved in processing current sensory information and encoding new memories. The fast gamma oscillations receive input from the medial entorhinal cortex to CA1, and these are involved in memory retrieval [15]. It appears that different frequencies in the entorhinal-hippocampal network are responsible for memory encoding and memory retrieval. These specific circuits can be identified through recording the different frequencies and following the frequency recording over time delineating the pathway in which information is relayed through the different anatomical locations. Recording all the frequencies and identifying the abnormal frequency suggest that stimulating at the specific frequency should affect one aspect, not all, of the memory deficits. Indeed, stimulation of the entorhinal cortex improved memory encoding in epilepsy patients, whereas slow gamma stimulation at 50 Hz of the hippocampus or entorhinal cortex impairs memory, possibly due to decreased ability to process current sensory information and encoding new memories [15-17]. In addition to the entorhinal-hippocampal network, the inhibitory interneuron network is critical for the production of gamma oscillations needed for memory retrieval [11]. Brain inflammation, oxidative stress, or metabolic imbalances can disrupt the network. Using a mouse model of genetically enhanced NF-κB activity (nfκb1-/-) that induces low-grade chronic inflammation and premature aging, the authors found deficits in gamma frequency oscillations along with early onset of memory loss, enhanced neuroinflammation, and increased frequency of senescent cells in the hippocampus. Treating these subjects with the nonsteroidal anti-inflammatory drug (NSAID) ibuprofen reduced neuroinflammation and senescent cell burden resulting in improvements in cognitive function and gamma frequency oscillations [18]. Abnormalities in gamma oscillations have been found in neurological disorders that involve memory impairments such as Alzheimer’s disease, Parkinson's disease, schizophrenia, and fragile X syndrome [11,19].

There are several neuromodulation techniques, such as repetitive transcranial magnetic stimulation (rTMS), transcranial alternating current stimulation (tACS), transcranial direct current stimulation (tDCS), and deep brain stimulation (DBS) that deliver gamma oscillations to synchronize disrupted neuronal networks, thus restoring gamma synchrony via regulating excitatory-inhibitory imbalances among different brain regions and improving cognitive and behavioral deficits [20]. In the brain, a superordinate cognitive control network, which involves dorsolateral prefrontal, anterior cingulate, and parietal cortices, governs a broad range of executive functions to exhibit complex, goal-directed behaviors [21]. Abnormal amyloid level disrupts frontal gamma synchronization with initial decrease in gamma activity followed by an increase in gamma activity correlating with accumulation of amyloid and then a decrease in gamma activity at the highest levels of amyloid [22]. Overall, gamma oscillations delivered via transcranial electrical stimulation, transcranial magnetic stimulation, and rhythmic sensory stimulation in mild cognitive impairment and AD patients improved performance on memory tests, increased gamma oscillatory responses, reduced brain atrophy, and delayed cognitive decline [23].

Several studies have found that gamma stimulation at 40 Hz enhanced cognitive function in Alzheimer’s disease [20]. This is different from the study by Jacobs et al., which showed that slowed gamma stimulation at 50 Hz of the hippocampus or entorhinal cortex impairs memory [17]. Thus, knowing the exact abnormal frequency is important to treat the patient. The gamma frequency band is roughly defined as being between 30 Hz and 100 Hz [24]. In Alzheimer's disease mouse models, studies have found that multisensory gamma stimulation at 40 Hz is effective in decreasing amyloid-β accumulation through suppression of its production, enhanced microglial clearance, promotion of cerebrospinal fluid movement via aquaporin-4 polarization, and increased arterial pulsation and lymphatic vessel expansion [25-27]. Supporting the findings in McDermott et al. that “the 40 Hz frequency value seems of particular neurological importance and as such represents a natural target value (with) promise for clinical application to AD,” more studies establish gamma neuromodulation as a promising treatment option for patients at risk of, or with, AD [24]. These studies found participants at risk of, or with, AD exhibiting improved cognitive (e.g., executive, attention, processing speed, semantic memory, verbal fluency), clinical (e.g., sleep, daily), and neurophysiologic (e.g., gamma) function, as well as improved bilateral hippocampal perfusion, and reduced ventricular enlargement, and loss of central nervous system (CNS) white matter (WM) [28-37]. Babiloni and colleagues found that the “relatively low sampling frequency” utilized in many studies precludes specific assessment of “EEG signal beyond 40 Hz,” suggesting that higher frequencies were not investigated [38]. Targeting specific abnormalities measured in a specific patient may provide quicker and more profound mitigation of symptoms.

However, higher frequency stimulation has been studied into the bilateral deep-brain fornix. 130 Hz stimulation here activates bilateral temporal (including hippocampal) and cingulate (including anterior cingulate) activity, as well as medial prefrontal activity. Stimulation of the hippocampus and medial prefrontal areas in AD patients via DBS to the fornix showed improvements in cognition and behavior [39]. Delivery of high frequency 130 Hz via DBS to the entorhinal-hippocampal network improves cognition through reducing inflammation and increasing synaptic proteins rather than facilitating rhythmic coordination of cells in AD [40].

These techniques (repetitive transcranial magnetic stimulation (rTMS), tACS, tDCS, and deep brain stimulation (DBS)) are not without their disadvantages, including invasiveness and transportation issues. For example, DBS is invasive and requires patients to undergo surgery. Patients may also need to be transported to a room designed with the right equipment for transcranial stimulation, and a shortage of staff to bring patients for treatment may limit the number of treatment sessions. Therefore, a non-invasive, portable, and light-weighted helmet with sensors was built to record human EMF and perform neuromodulation via stimulation of the human brain with specific frequencies [41-44]. Our innovation has been used to modulate abnormal EMF patterns and improve clinical symptoms and signs in patients with a variety of neurological diseases, including concussion, stroke, traumatic subarachnoid hemorrhage, spontaneous intracranial hemorrhage, and neoplasm [45-49]. Our technique of delivering EMF stimulation is unique. First, it delivers both electric and magnetic fields to the brain, not just magnetic like rTMS. Second, our EMF stimulation is delivered without direct contact with the patient’s scalp; no electrodes are used, unlike in tACS. Third, deep brain stimulation is invasive; ours is not.

Here, we describe a non-invasive neuromodulation technique via electromagnetic field stimulation at higher gamma oscillations, specifically from 70 Hz to 100 Hz, in patients with cognitive impairment and utilize the Mini-Mental State Examination scores to evaluate its effects on cognition and memory.

## Materials and methods

Study design 

This was a prospective study approved by the institution's institutional review board (Protocol #23-58: Transcranial electromagnetic field stimulation for modulation of brain activity in patients with neurological disorders). From April 2025 to September 2025, we included patients over 18 years with history of memory issues, dementia, or Alzheimer's disease provided by the patient's relatives. We excluded intubated patients or subjects with limitations to participate in the study and undergo the study intervention. MMSE was performed to identify patients with dementia followed by baseline EMF recording, which was analyzed to propose a methodology of identifying the sensor of interest and frequency of interest in six patients with cognitive impairment. Then, ten new patients underwent pre-stimulation MMSE testing followed by pre-stimulation EMF recording, EMF stimulation, post-stimulation EMF recording, and post-stimulation MMSE testing. 

Mini-Mental State Examination (MMSE)

The findings from MMSE were categorized into specific cognitive functions including orientation, registration, attention and calculation, recall, and language and praxis. An unauthorized version of the English MMSE was used by the study team without permission, however this has now been rectified with PAR. The MMSE is a copyrighted instrument and may not be used or reproduced in whole or in part, in any form or language, or by any means without written permission of PAR (www.parinc.com).

Electromagnetic field (EMF) recording

We used our previously designed helmet and sensors (Table 1) [46-49]. In brief, two portable racks equipped with horizontal rods and four elastic cords were used to suspend the light-weight helmet in air, just above the patient’s head. The portable helmet with shielding constrained to a dual-layered Mu-metal (MuMETAL, Magnetic Shield Corporation, Bensenville, IL) and copper layering and engineered with Mu-metal 18-inch channels to place sensors and EMF signal generators (BS-1000, Quasar Federal Systems, San Diego, CA) was built to measure EMF recording and deliver EMF stimulation. The known spatial relationship between the sensors allowed for identifying regions of overlap or opposite configurations, where sensors in opposing positions (180° from each other) were expected to demonstrate opposite polarities for a specific EMF. EMF recordings were collected using the DAQami software (Dataq Instruments, Akron, OH) and analyzed using fast Fourier transformation (FFT) with the Igor Pro 8 software (Wavemetrics Inc., Lake Oswego, OR). Each EMF recording was 30 seconds. To ensure stability of data, only 20 seconds of the recording were used for analysis (data between five seconds after the start of the recording and five seconds before the end of the recording). Graphs of EMF recordings were then generated using FFT with the Igor Pro 8 software. EMF signal generators (BS-1000, Quasar Federal Systems, San Diego, CA) were used to deliver various voltage stimulations at the targeted frequency of interest (FOI). A sine wave was used in this study.

**Table 1:** Electromagnetic sensors and their corresponding brain cortex regions.

Sensor	Corresponding brain regions
1	Bifrontal
2	Right frontal
3	Right parietal
4	Biparietal
5	Left parietal
6	Left frontal
7	Right frontal
8	Right frontotemporal
9	Right anterior temporal
10	Right middle temporal
11	Right posterior temporal
12	Right parietooccipital
13	Right occipital
15	Left occipital
16	Left parietooccipital
17	Left posterior temporal
18	Left middle temporal
19	Left anterior temporal
20	Left frontotemporal
21	Left frontal

Identification of sensor of interest and frequency of interest 

A total of 16 patients were included in this prospective study. EMF recordings from six patients helped formulate the protocol on how to analyze the data. These six patients did not undergo EMF stimulation. The other ten patients completed the study protocol. The first six patients undertook the MMSE testing and all of them had cognitive impairment (score < 24). The specific cognitive deficits found via MMSE testing corresponded to specific brain regions and thus helped to localize focusing on specific sensors of interest that overlie these brain regions. Many sensors of interest were identified from MMSE testing. Next, a baseline EMF recording was performed and analyzed. Previously, localization of the worst brain injury and its corresponding sensor of interest was determined from clinical presentation (for example, headache in the right frontal region, which corresponds to sensor 7) [46]. However, given that cognitive impairment is global and multiple areas are affected, there are multiple corresponding sensors and thus it becomes difficult to determine the sensor of interest that corresponds to the brain area with the most deficits. Therefore, the derivatives of baseline EMF recordings were analyzed. Previously, the derivatives of EMF in healthy individuals and patients with neurological diseases showed overall less defined morphology, with a much flatter waveform (fewer peaks and valleys) and more negative slopes in frequencies less than 2 Hz. The frequency range from 0.0 to 4.0 Hz was found to be most affected in brain-damaged people [45]. Here, the first- and second-derivative graphs were generated, and the sensor with the lowest amplitude in 0.1-12 Hz was selected as the sensor of interest, reflecting the brain region with the most deficits. This sensor of interest was then used to identify the frequency of interest to be stimulated at and evaluate the effect of EMF stimulation on neuromodulation. Then, the sensor of interest and its opposing sensor were isolated on the original EMF recording from 0 Hz to 100 Hz. The frequency at which the sensor of interest showed a valley and the opposing sensor showed a peak was selected as the frequency of interest. There may be multiple frequencies of interest; the frequency with the lowest amplitude was selected for EMF stimulation. 

Electromagnetic field (EMF) stimulation

The effect of EMF stimulation on patients with cognitive impairment was studied in ten new patients. First, pre-stimulation MMSE testing was administered to screen for cognitive impairment consistent with dementia. Next, a pre-stimulation EMF recording was performed and analyzed to identify the sensor of interest and frequency of interest as described above. Then, each patient underwent EMF stimulation at the frequency of interest at 10 V over 10 minutes. Previously published studies by the authors demonstrated no untoward effects at those parameters. Furthermore, this was the limit of the current equipment. Afterward, post-stimulation EMF recording was performed. Finally, the patients underwent post-stimulation MMSE testing. Pre- and post-MMSE scores and pre- and post-EMF stimulation recordings were compared.

## Results

A total of 16 patients were included in this pilot study. First, a total of six patients' MMSE findings and EMF recordings (their derivatives) were used to localize six individualized sensors of interest. All six identified sensors of interest and their opposing sensors showed a pair of valleys and peaks, respectively, between 70 Hz and 100 Hz (data not shown). The way the data were analyzed in these six patients became the established methodology. 

**Figure 1:**
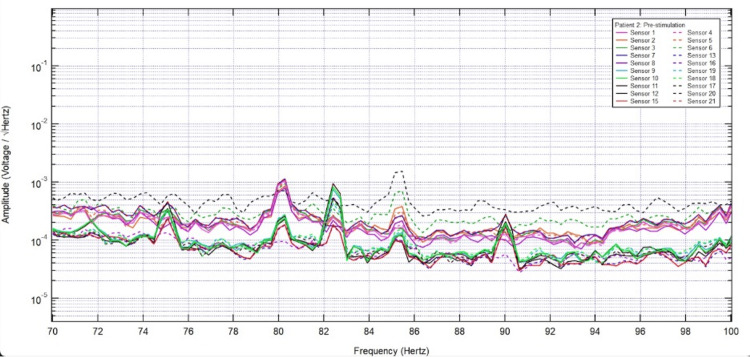
Pre-stimulation electromagnetic field recording of all twenty sensors from 70 Hz to 100 Hz.

**Figure 2:**
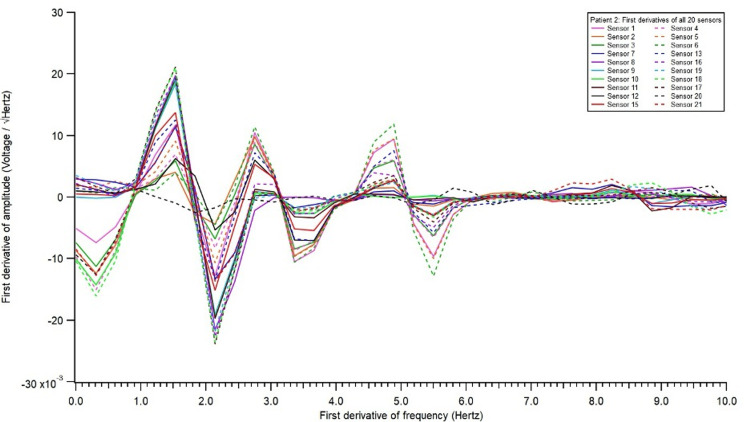
Electromagnetic field recording of first derivatives of all 20 sensors.

**Figure 3:**
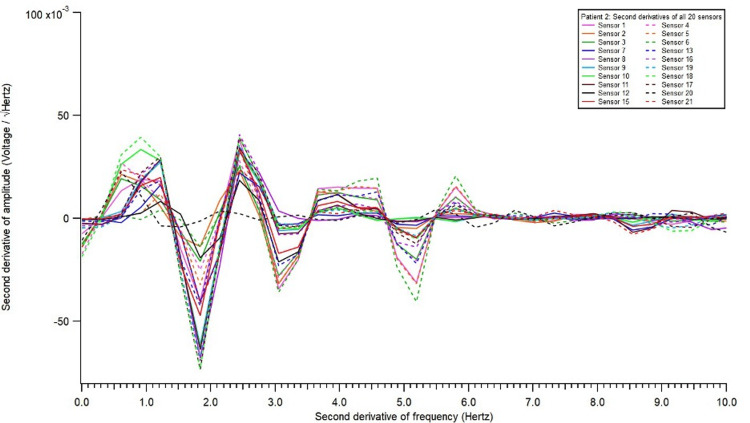
Electromagnetic field recording of second derivatives of all 20 sensors.

**Figure 4:**
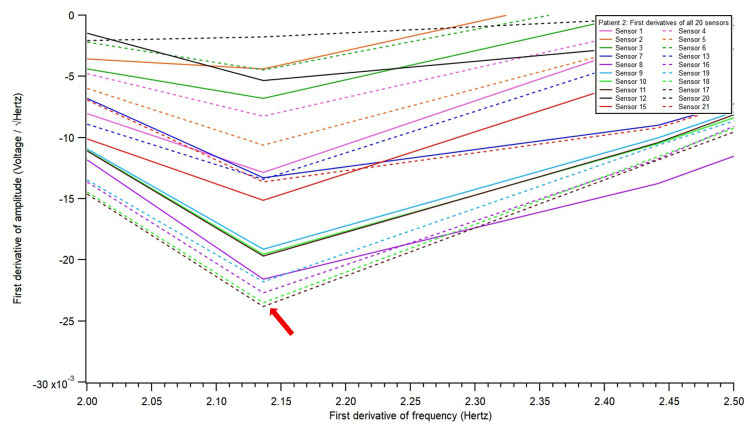
A closed up look at the electromagnetic field recording of first derivatives of all 20 sensors at the point of lowest amplitude, as defined in the Methods section. Identification of sensor of interest and frequency of interest. The sensor with the lowest y-axis value was sensor 17 (red arrow); therefore, it was identified as the sensor of interest. Nearby sensors, 16 and 18, also showed similar derivative patterns.

**Figure 5:**
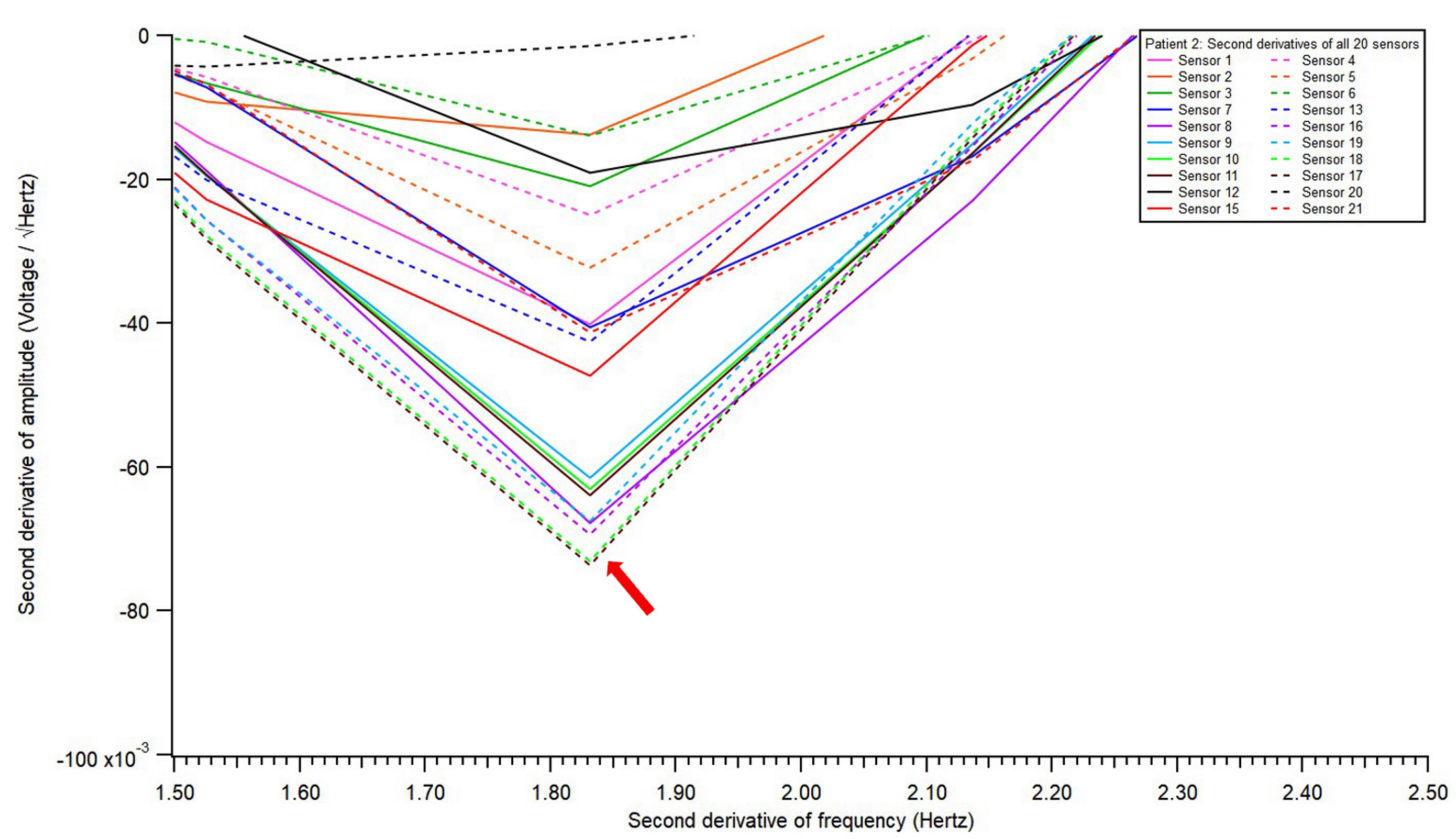
A closed look at the electromagnetic field recording of second derivatives of all 20 sensors at the point of lowest amplitude. The sensor with the lowest y-axis value again was sensor 17 (red arrow); therefore, sensor 17 was confirmed as the sensor of interest. Sensor 18, which was in a closer proximity to sensor 17, showed an overlapping derivative pattern; however, sensor 16, which was farther away from sensor 17, showed a similar but nonoverlapping pattern.

**Figure 6:**
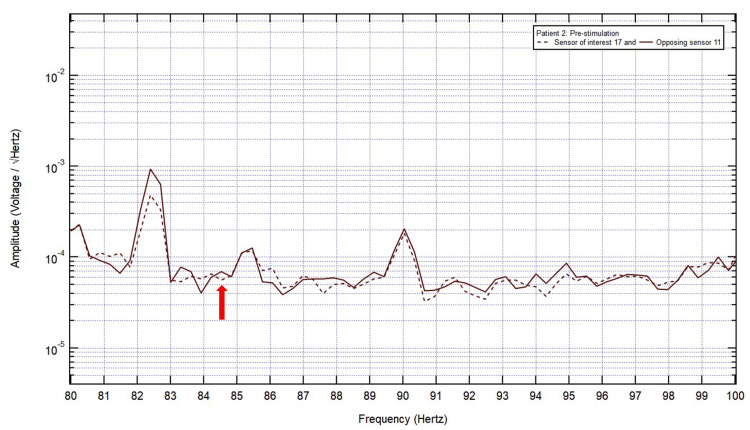
After identifying sensor 17 as the sensor of interest, sensor of interest 17 and the opposing sensor 11 were isolated from other sensors. Frequencies from 0 to 100 Hz were analyzed to help identify the frequency of interest. A valley in the sensor of interest 17 and a peak in the opposing sensor were found at 84.5 Hz (red arrow); therefore, it was identified as the frequency of interest.

**Figure 7:**
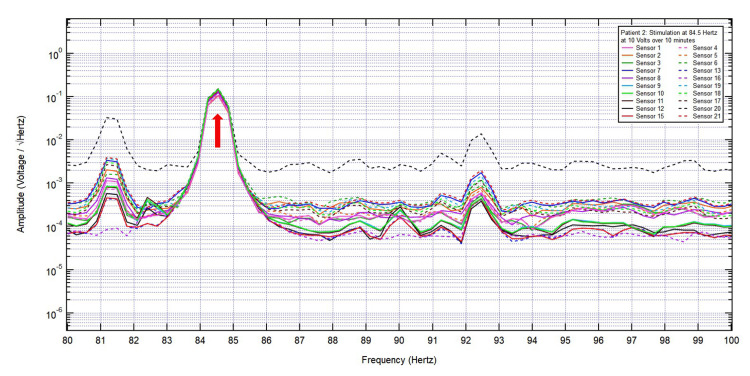
Stimulation of all 20 sensors at the frequency of interest 84.5 Hz (red arrow) at 10 V over 10 minutes.

**Figure 8:**
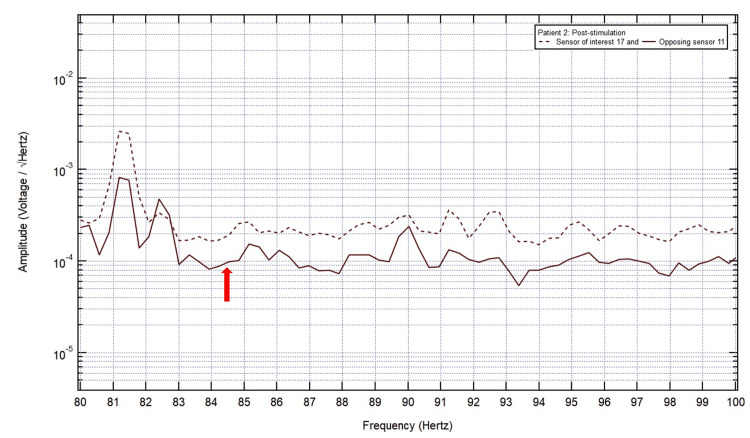
Post-stimulation electromagnetic field recording showed a positive slope at 84.5 Hz (red arrow) in sensor of interest 17.

Next, a total of 10 patients underwent MMSE testing and EMF stimulation. The mean age was 82.9 years (range 67-91 years). There were six female patients and four male patients. The mean pre-stimulation MMSE score was 13.8/30 points, and the post-stimulation MMSE score was 17.5/30 points (p=0.170). In each category, the pre-stimulation MMSE score and the post-stimulation MMSE score were: (1) orientation: pre 3.3/10 points vs. post 5.0/10 points (p=0.162); (2) registration: pre 2.5/3 points vs. 2.6/3 points (p=0.795); (3) attention and calculation: pre 0.6/5 points vs. 0.7/5 points (p=0.791); (4) recall: pre 0.8/3 points vs. 1.7/3 points (p=0.112); and (5) language and praxis: pre 6.6/9 points vs. 7.5/9 points (p=0.377). The specific areas of cognitive deficits are shown in Table 2. The first and second derivatives identified the sensor of interest overlying the brain region with the most deficit, and these sensors of interest, with their corresponding brain area, are listed in Table 3. The frequencies of interest are listed in Table 2. The frequencies of interest with the lowest amplitude were stimulated at 76.0 Hz, 82.4 Hz, 84.5 Hz, 86.1 Hz, 89.1 Hz, 89.4 Hz, 92.8 Hz, 94.6 Hz, 97.7 Hz, and 98.0 Hz, and these frequencies were stimulated at 10 V over 10 minutes. Additionally, post-stimulation EMF recordings showed peaks (n=2 patients), positive slope (n=4 patients), and plateau (n=4 patients) at the frequency of interest. Patient 2 is used as an illustrated example (Figures 1-8). 

**Table 2:** Performance on Mini-Mental State Examination of 10 patients who underwent electromagnetic field stimulation.

Cognitive function (total points) and its corresponding brain regions	Patient 1		Patient 2		Patient 3		Patient 4		Patient 5		Patient 6		Patient 7		Patient 8		Patient 9		Patient 10	
	Pre-stim	Post-stim	Pre-stim	Post-stim	Pre-stim	Post-stim	Pre-stim	Post-stim	Pre-stim	Post-stim	Pre-stim	Post-stim	Pre-stim	Post-stim	Pre-stim	Post-stim	Pre-stim	Post-stim	Pre-stim	Post-stim
Orientation (10 points) Medial prefrontal frontal, temporal, posterior cingulate cortex, fusiform gyrus	2/10	4/10	1/10	3/10	1/10	2/10	0/10	2/10	7/10	8/10	7/10	8/10	6/10	7/10	5/10	8/10	2/10	3/10	2/10	5/10
Registration (three points) Wernicke's, Broca, arcuate fasciculus	3/3	3/3	3/3	3/3	1/3	1/3	1/3	1/3	3/3	3/3	3/3	3/3	3/3	3/3	2/3	3/3	3/3	3/3	3/3	3/3
Attention and calculation (five points) Prefrontal, frontal dorsolateral, frontal, inferior parietal, cingulate gyrus, left parietal	1/5	1/5	0/5	0/5	0/5	0/5	0/5	0/5	2/5	2/5	0/5	1/5	1/5	1/5	2/5	2/5	0/5	0/5	0/5	0/5
Recall (three points) Hippocampus, medial temporal lobe	0/3	0/3	0/3	0/3	2/3	3/3	0/3	1/3	0/3	3/3	2/3	3/3	1/3	3/3	1/3	1/3	0/3	0/3	2/3	3/3
Language and praxis (nine points) Wernicke's, Broca's, fasciculus arcuatus, temporal, prefrontal, frontal, premotor, parietal, occipital, basal ganglia	9/9	9/9	8/9	8/9	2/9	3/9	3/9	4/9	8/9	9/9	7/9	8/9	7/9	9/9	8/9	9/9	7/9	8/9	7/9	8/9
30 points total	15/30	17/30	12/30	14/30	6/30	9/30	4/30	8/30	20/30	25/30	19/30	23/30	18/30	23/30	18/30	23/30	12/30	14/30	14/30	19/30

**Table 3:** Results from electromagnetic field recordings. The mean pre-stimulation MMSE score was 13.8/30 points and the mean post-stimulation MMSE score was 17.5/30 points (p=0.170), showing a trend toward cognitive and memory improvement without statistical significance. EMF: electromagnetic field; MMSE: Mini-Mental State Examination.

Patient number	Age in years	Sex	Sensor of interest	Location of sensor of interest	Frequencies of interest (FOI) in hertz (Hz)	FOI stimulated	Post-stimulation EMF at FOI	Pre-stimulation MMSE score	Post-stimulation MMSE score
Patient 1	91	Female	13	Right occipital	28.4, 41.8, 75.8, 92.8	92.8 Hz	Plateau	15/30	17/30
Patient 2	91	Male	17	Left temporal	25.6, 84.4	84.5 Hz	Positive slope	12/30	14/30
Patient 3	67	Female	7	Right frontal	36.9, 89.4	89.4 Hz	Positive slope	6/30	9/30
Patient 4	86	Female	5	Left parietal	98.0	98.0 Hz	Plateau	4/30	8/30
Patient 5	88	Male	7	Right frontal	82.4	82.4 Hz	Peak	20/30	25/30
Patient 6	83	Female	12	Right parietal	89.1	89.1 Hz	Peak	19/30	23/30
Patient 7	84	Male	10	Right temporal	32.6, 44.3, 88.4, 97.7	97.7 Hz	Positive slope	18/30	23/30
Patient 8	87	Female	13	Right occipital	48.2, 57.4, 79.6, 94.6	94.6 Hz	Plateau	18/30	23/30
Patient 9	73	Female	12	Right parietal	40.6, 68.7, 86.1	86.1 Hz	Plateau	12/30	14/30
Patient 10	79	Female	1	Bifrontal	76.0	76.0 Hz	Positive slope	14/30	19/30

## Discussion

This pilot study suggests possible cognitive and memory improvements via neuromodulation of human brain EMF at higher gamma oscillations, specifically 70 Hz to 100 Hz, as assessed with the MMSE in patients with cognitive impairment. To the best of the authors’ knowledge, this is the first study to investigate the effects of stimulation from 70 Hz to 100 Hz on cognition and memory performance. From the first and second derivatives, the brain region with the most deficits was identified as the corresponding sensor of interest that showed the lowest amplitude. This sensor was defined as the sensor of interest, and the frequency of interests were then identified. 

EMF stimulation leads to changes in EMF patterns. Post-EMF recordings of all 10 patients showed there was no valley at the frequency of interest. The valley became a plateau, a positive slope, and a peak. The varieties of changes in EMF patterns suggest that different voltages may be necessary to transition a valley into a peak, as evident that some valleys became peaks after a 10 V stimulation, while others did not. Similar findings were found on post-EMF stimulation recordings in patients with traumatic brain injury [48]. Unfortunately, 10 V is the maximum voltage that can be delivered via the EMF stimulator. These EMF changes are interpreted as a reflection of molecular, cellular, and circuit changes that translate into clinical improvement [46-51].

Despite not having a transformation from a valley to a peak, all patients showed improved MMSE scores, suggesting improved cognition and memory. Even though there is a possibility of learning the questions and answers on MMSE, the interval between the two MMSEs was over an hour. Therefore, the improved MMSE scores seen on post-stimulation MMSE are less likely due to learning and more likely due to neuronal changes, as evident by the changes in EMF patterns. This finding of improved EMF patterns seen with improved clinical symptoms and signs is consistent with our prior study’s findings [48]. Previously, a maximum of 10 minutes of EMF stimulation was administered, resulting in clinical improvement [49]. In this study, all 10 patients underwent 10 minutes of EMF stimulation with improved scores on post-EMF stimulation recording. Further studies should investigate whether longer, repeated stimulation would further improve cognition and memory, as assessed with a cognitive screening tool such as the MMSE. Additionally, a control or sham group in future studies may help confirm effect specificity. 

Previously, studies have found gamma neuromodulation at 40 Hz in patients at risk of or with AD showed improved cognitive (e.g., executive, attention, processing speed, WM, semantic memory, verbal fluency), clinical (e.g., sleep, daily), and neurophysiologic (e.g., gamma) function, as well as improved bilateral hippocampal perfusion, and reduced ventricular enlargement, and loss of CNS white matter [24,28-37]. In our study of the 10 patients who underwent EMF stimulation, 5/10 patients (50%) showed frequencies of interest between 30 Hz and 50 Hz (Table 2). Patient number 1 had Alzheimer’s disease and a low amplitude at 41.8 Hz. These EMF findings may correlate with more amyloid deposits. There may be a threshold in the level of amyloid deposits before changes can be detected on EMF recordings; thus, not all patients in this study showed frequencies of interest between 30 Hz and 50 Hz. The discrepancy may be due to the differences in methodology and heterogeneity of the patients studied. The 10 patients in this study were found to have cognitive impairment based on MMSE, and the specific pathology, such as AD underlying each patient’s cognitive impairment, was not diagnosed at the time of this study, except for patient 1. The 40 Hz frequency change cited above was identified in Alzheimer’s patients and has not been described in other patients. The only patient in our study who had been diagnosed with Alzheimer’s Disease had changes at 41.8 Hz. Our methods allow specific identification of brain EMF in specific anatomical locations; it overcomes the deficiency of other methods, which group patients and neuronal circuits. Amyloid deposits are found in patients with AD, and not all cognitively impaired patients have a buildup of amyloid. However, all 10 patients appear to have deficits in synchronization and memory retrieval based on their MMSE performances, especially in orientation, attention and calculation, recall, and language and praxis. This study shows that 40 Hz is not the only frequency of interest in cognitive deficit patients and future studies should take into consideration performing neuromodulation at 70-100 Hz and individualizing patients and their deficits. Frequencies higher than 100 Hz were not analyzed in this study, given the study’s design. Previous studies have found DBS stimulation at 130 Hz to the fornix and the entorhinal-hippocampal network improves cognition through reducing inflammation and increasing synaptic proteins [39,40]. Future studies should investigate other frequencies and their role in cognition, memory, and emotion. 

Cognitive impairment is a global brain phenomenon as evident by global brain atrophy [4-10]. Some brain regions show more atrophy than others, which can be explained by the severity of cognitive and memory deficits. Multiple sensors spanning all four lobes (frontal, parietal, temporal, and occipital) in both hemispheres were found to be sensors of interest, suggesting that cognitive impairment is not specific to one brain region but rather it is global and due to disruptions of neuronal circuits that make up a superordinate cognitive control network involving various brain regions to govern and carry out tasks focusing on orientation, registration, recall, language, and praxis (all the categories tested in the MMSE) [21]. Indeed, gamma oscillations likely allow for re-synchronization of these disrupted neuronal networks, thus restoring gamma synchrony via regulating excitatory-inhibitory imbalances among different brain regions and improving cognitive and behavioral deficits [20]. Higher frequencies, such as between 70 Hz and 100 Hz, may present deficits in synchronization and memory retrieval, while 40 Hz may represent deficits in processing current sensory information and forming new memory. Thus, EMF stimulation targeting these higher frequencies restore normal synchronization, which may underlie improved drawing and writing and overall scores on MMSE. It is important to understand the EMF deficit of the person and how it relates to clinical signs and symptoms and stimulate at that specific frequency.

This study provides preliminary evidence that EMF stimulation may modulate brain activity in cognitive disorders. Previously, the same equipment used in this study was used on patients with concussion, stroke, traumatic subarachnoid hemorrhage, spontaneous intracranial hemorrhage, and neoplasm and neuromodulation via EMF stimulation was effective in improving abnormal EMF patterns and their clinical symptoms [45-49]. Here, this study shows successful neuromodulation via EMF stimulation on patients with cognitive impairment. It would be of utmost interest to study the effect of EMF stimulation on patients with Alzheimer’s disease on cognition and memory long-term given the burden of the disease on the patient, his/her family, and society. 

Limitations 

This is a pilot study with a small sample size and no short-term or long-term follow-up. This could explain the lack of statistical significance, while clinical significance in cognitive function improvement was achieved. Also, there is no control or sham group to compare, thus limiting causal interpretation. There is also a lack of blinding in this study. The patient population of this study was heterogenous in respect to the level of cognitive impairment based on MMSE scores. Perhaps, multiple cognitive screening tests can be utilized to better categorize patients on the spectrum of cognitive impairment, which is a complex and progressive disease process. Future studies should focus on specific subsets of patients with cognitive impairment, investigate the specific pathophysiology in each subset, and evaluate the effect of neuromodulation on cognition and memory for each unique patient population during short-term and long-term stimulation.

## Conclusions

Cognitive impairment is on the rise and its associated burden on the patient and society is unmeasurable. Screening tools, such as MMSE, have been used to identify people with cognitive impairment. Neuromodulation of abnormal gamma stimulation at 40 Hz and 130 Hz enhances cognitive function in different subtypes of patients with cognitive disease specific to the patient and their disease process. Here, this pilot study shows that EMF stimulation between 70 Hz and 100 Hz tailored to the specific abnormality can increase brain EMF wave amplitude and improve MMSE scores in multiple subtypes of patients with cognitive disease, suggesting that neuromodulation at these frequencies can help improve cognition, memory, writing, and drawing via restoring normal synchronization of various brain regions and circuits. Specifically, neuromodulation via EMF can facilitate processing stimuli, create new memories, and, most importantly, synchronize the information to retrieve information at a later time point. The findings in this study are preliminary and will require validation in larger, randomized, controlled studies in specific patient populations and long-term follow-ups.
